# Identifying Chinese Herbal Medicine Network for Eczema: Implications from a Nationwide Prescription Database

**DOI:** 10.1155/2015/347164

**Published:** 2015-01-22

**Authors:** Hsing-Yu Chen, Yi-Hsuan Lin, Sindy Hu, Sien-hung Yang, Jiun-liang Chen, Yu-Chun Chen

**Affiliations:** ^1^Division of Chinese Internal Medicine, Center for Traditional Chinese Medicine, Chang Gung Memorial Hospital, Taoyuan 33378, Taiwan; ^2^Graduate Institute of Clinical Medical Sciences, College of Medicine, Chang Gung University, Taoyuan 33302, Taiwan; ^3^School of Traditional Chinese Medicine, College of Medicine, Chang Gung University, Taoyuan 33302, Taiwan; ^4^Department of Cosmetic Science, Chang Gung University of Science and Technology, Taoyuan 33302, Taiwan; ^5^Department of Dermatology, Chang Gung Memorial Hospital, Taoyuan 33378, Taiwan; ^6^Department of Medical Research and Education, National Yang-Ming University Hospital, Ilan 26042, Taiwan; ^7^Institute of Hospital and Health Care Administration, School of Medicine, National Yang-Ming University, Taipei 112, Taiwan

## Abstract

Eczema is a highly prevalent dermatological disease that can severely affect the patient's quality of life. Chinese herbal medicine (CHM) is commonly used in combination for eczema due to the complicated pathogenesis. This study aimed to identify a CHM network for the treatment of eczema by using a nationwide database. During 2011, 381,282 CHM prescriptions made for eczema (ICD-9-CM 692.x) were obtained from the National Health Insurance Research Database (NHIRD) in Taiwan and analyzed by using association rule mining and social network analysis. Among 661 available CHMs, 44 important combinations were identified. Among the CHM networks, seven clusters with the predominant traditional Chinese medicine (TCM) pattern were recognized. The largest CHM cluster was used to treat the wind-dampness-heat pattern, and Xiao-Feng-San (24.1% of all prescriptions) was the core of this cluster with anti-inflammation, antioxidation, and antiallergic effects. *Lonicera japonica* (11.0% of all prescriptions) with *Forsythia suspense* (17.0% of all prescriptions) was the most commonly used CHM combination and was also the core treatment for treating the heat pattern, in which an antimicrobial effect is found. CHM network analysis is helpful for TCM doctors or researchers to choose candidates for clinical practice or further studies.

## 1. Introduction

Eczema is a prevalent chronic inflammatory dermatological disease, with an increasing prevalence and financial burden, especially in Asian countries [1, 2]. In addition to being highly associated with other comorbidities, eczema is characterized by frequent relapses of skin itching, erythema, scratching, and discharge and thus can severely affect the patients' quality of life [1, 3]. While there is no definite cure for eczema, antihistamines, corticosteroids, emollients, and even immune-modulating agents are often used concurrently to control symptoms. Corticosteroids are one of the most important and effective treatments for eczema caused by a Th2 dominant immune response [4, 5]. However, recurrent symptoms of eczema commonly lead to the prolonged use of corticosteroids which can cause side effects, including skin atrophy and angioectasia that can easily lead to bleeding, immunosuppression, euphoria, and endocrine dysfunction [6, 7]. Due to the inadequacy of current treatment, finding an optimal treatment strategy to treat eczema without long-term complications remains an urgent issue [4, 8].

Traditional Chinese medicine (TCM) is one of the most commonly used alternative medicines in Taiwan due to the perceived favorable efficacy with fewer side effects [9, 10]. Increases in the cost and use of TCM including Chinese herbal medicine (CHM), acupuncture, and massage have been reported in recent years. With regard to treatment for eczema, CHM is much more commonly used than other modalities of TCM [11]. Despite the high prevalence of the use of CHM, the composition of CHM prescriptions for eczema is unclear and such investigations are lacking. Analyzing these prescriptions is important to clarify the role CHM may play in the treatment of eczema and also in the selection of suitable candidates for clinical trials and bench studies, especially when the CHM used in daily practice may be different from classic TCM [12, 13]. In addition, the high heterogeneity in CHM reported in meta-analyses of systematic reviews may be related to a lack of information on the CHM prescriptions used in clinical practice [11, 13].

A CHM network is helpful in understanding the rationale of CHM prescriptions through a graphical demonstration of relationships between CHMs [14]. Network analysis is especially important when trying to understand how prescriptions are made, since TCM doctors frequently combine five to six CHMs in one prescription from hundreds of available CHMs [15, 16]. The choice of the CHMs is mainly dependent on the characteristics of the other CHMs, such as four qi and five flavors, in the same prescription. Specific connections between pairs of CHMs, such as herbal pairs, are the key components of CHM prescriptions, and thus a sophisticated CHM network can be formed [14, 17]. In addition, further studies on the efficacy and active ingredients may be facilitated on the basis of a CHM network [18–20].

This study aimed to identify a CHM network for the treatment of eczema, composed of commonly used CHMs and combinations by analyzing a nationwide prescription database. These findings are not only helpful in knowing the rationale of CHM formulation but also beneficial to further studies when selecting their candidates.

## 2. Material and Methods

### 2.1. Data Source

A cross-sectional CHM prescription dataset obtained from the National Health Insurance Research Database (NHIRD), Taiwan, was used in this study. The National Health Insurance (NHI) program was established in Taiwan in 1995 and currently provides comprehensive medical care for over 99% of the 23-million population. All information required for NHI reimbursement, including gender, birth date, reasons for visits and interventions (including medication, frequency, and duration), examinations ordered by the doctors, prescriptions, and expenses, is digitized and stored in the NHIRD. The patient's identification number is encrypted in the NHIRD, and thus an individual's true identity cannot be traced. To verify the reason for each visit, up to three diagnoses are recorded using International Classification of Diseases, Ninth Revision, Clinical Modification (ICD-9-CM) codes. Furthermore, the first diagnosis code is required to be the main reason for each visit. The reliability of ICD-9-CM coding as reasons for visits has been proven, and the NHIRD itself has been successfully used for many studies including core treatments from prescription analysis and analysis of the characteristics of patients receiving TCM [10, 21, 22]. In addition, the utilization of CHM is unique in Taiwan. Reimbursements for both CHM and western medicine (WM) are made equally, and therefore patients are free to choose treatments without bias. Due to the high coverage of the NHI, the results of analysis using prescriptions can be seen as a general consensus, and potential selection or referral bias can therefore be effectively eliminated [16, 22].

### 2.2. Study Subjects

To identify visits made for TCM treatments for eczema, a single diagnosis of eczema (ICD-9-CM code: 692.x) was used. Eczema consists of a range of persistent dermatologic manifestations, including skin pruritus, erythema, swelling, and lichenification with prominent scratching. Visits with a first ICD-9-CM code other than 692.x or other ICD-9-CM codes in the second or third diagnosis were excluded to minimize potential confounding bias on the prescription caused by comorbidities.

### 2.3. Chinese Herbal Medicine Prescription Dataset

All visits with CHM prescriptions for eczema during 2011 were used to construct the CHM prescription dataset. Herbal formulas (HF) and single herbs (SH) are the only kinds of CHM reimbursed by the NHI in Taiwan. SH, such as* Angelica sinensis *(Dang Gui), include herbs, animal parts, and minerals recorded in TCM materia medica while HF, such as Xiao-Feng-San (XFS), are composed of several SH with fixed proportions according to TCM classics. HF and SH are processed into a concentrated powder form, and TCM doctors are able to freely combine multiple HF or SH into one prescription to achieve the therapeutic goals.

### 2.4. Statistical Analysis

Applying association rule mining (ARM) with social network analysis (SNA) on large-scale prescription datasets is helpful in exploring CHM networks, as we previously reported [15, 16]. ARM is used to identify commonly used CHMs and their combinations, while the CHM network can be established by incorporating CHM combinations in SNA. ARM is one of the most commonly used data-mining techniques and has been used extensively to explore relationships between study targets such as combinations of CHMs, coprescriptions of WM drugs, comorbidities of diseases, and TCM syndrome [23–27]. Two decisive factors, support factor and confidence factor, are used in the ARM model to explore commonly used CHMs and CHM combinations. Support factor represents the prevalence of an individual CHM, and confidence factor represents the strength of connection between CHMs. A higher support factor of a CHM means that the CHM is used more commonly, while a higher level of confidence factor between two CHMs means the relationship is closer. The detailed algorithm was described in our previous work [28]. The thresholds of support and confidence factors were set to 1% and 30%, respectively, according to our experience in the analysis of CHM for urticaria [15].

The relationships between CHMs are illustrated by SNA, which is a powerful analytic method to present a sophisticated CHM network by using the hierarchical cluster method [16, 28, 29]. Several measurements are used to characterize the CHM network, including modularity, degree, and centrality [30, 31]. Degree represents the frequency of connections to a certain CHM, and modularity represents the fitness of CHM clustering. Once the best modularity has been acquired, CHMs with closer relationships are classified as one cluster with relationships of different clusters being as distant as possible. The detailed algorithm was reported in a previous study [30]. This graphical presentation of CHM combination patterns is helpful to understand the role of CHM in treating various diseases, including eczema. The open-source software “R” with the “arule” package was used to obtain important CHM combinations and visualize the CHM network in this study.

## 3. Results

**Figure 1:**
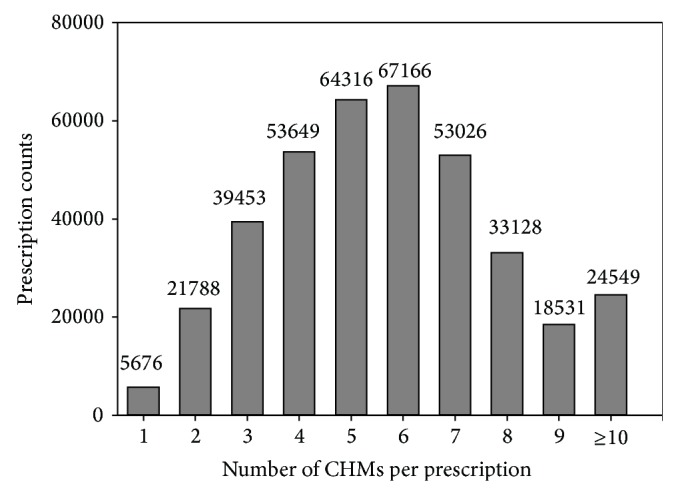
Distribution of the number of prescriptions.

There were 381,282 CHM prescriptions made for 132,971 eczema patients in 2011. Most of the CHM users were female (62.6%), most were aged between 21 and 30, and more than two-thirds were adults (Table 1). A total of 661 CHMs, including SH and HF, were used, and an average of approximately 5.8 CHMs was used per prescription on average. Multiple CHMs were commonly used in one prescription, and more than 90% of the prescriptions were composed of at least two CHMs (Figure 1).

Of the 661 available CHMs used to treat eczema in the CHM prescription dataset, the top 10 most commonly used CHMs (including SH and HF) are shown in Tables 2 and 3. XFS was the most commonly used HF (24.1%), followed by Qing-Shang-Fang-Feng-Tang (14.9%), Xian-Fang-Huo-Ming-Yin (12.5%), and Huang-Lian-Jie-Du-Tang (10.9%) (Table 2). Among all SH,* Forsythia suspensa* (Lian Qiao) was most commonly used (17.0%), followed by* Coix lacryma-jobi* (Yi Yi Ren) and* Taraxacum mongolicum* (Pu Gong Ying), accounting for 12.7% and 12.1% of all prescriptions, respectively (Table 3). The TCM doctors usually prescribed HF at three to four times higher dosage than SH, at about 4 gm/day for HF and 1–1.5 gm/day for SH (Tables 2 and 3).

Forty-four important CHM combinations were investigated in this study, the top 10 most common of which are listed in Table 4.* Lonicera japonica* (Jin Yin Hua) with* Forsythia suspensa* (Lian Qiao) was the most common combination (5.0% of all prescriptions), followed closely by XFS with* Dictamnus dasycarpus* (Bai Xian Pi) (4.8% of all prescriptions) (Table 4). XFS seemed to have an important role in treating eczema since it was used in 4 out of 10 combinations. In addition, the importance of its role in treating eczema was clearly shown when all significant combinations of CHMs were clustered by SNA.

**Figure 2:**
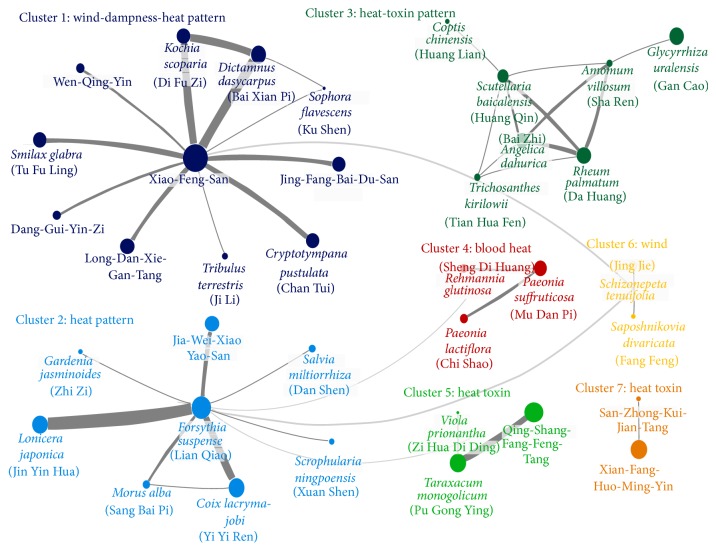
Chinese herbal medicine network for eczema.

Seven clusters were found in the CHM network for treating eczema by analyzing all CHM combinations as illustrated in Figure 2, in which larger circles and thicker connection lines represented a higher prevalence of the CHM and the combination, respectively. Each cluster had a predominant TCM syndrome as indicated when applying the characteristics of each CHM to the network; for example, the CHMs for wind dampness and heat syndrome were predominantly grouped in cluster 1 (Figure 2). CHMs in the same cluster had strong interconnections, while some clusters were used in combination, for example, cluster 1 (wind-dampness-heat syndrome) with cluster 6 (wind syndrome) and cluster 2 (heat-toxin syndrome) with clusters 4 (blood heat), 5 (heat toxin), or 6 (wind). XFS was at the center of the biggest cluster in the CHM network (Figure 2).

The pharmacological mechanisms of CHM were then accessed in a PubMed search and are summarized in Table 5 (last accessed date: 2014/6/30). Antioxidation, anti-inflammation, and antiallergic effects were frequently found among the commonly used CHMs. However, XFS was the only CHM to reportedly have all the effects. Interestingly, antimicrobial effects were reported in some of the CHMs, including Huang-Lian-Jie-Du-Tang,* Forsythia suspensa* (Lian Qiao), and* Lonicera japonica *(Jin Yin Hua).

## 4. Discussion

To the best of our knowledge, this is the first nationwide study to investigate a CHM network for the treatment of eczema. In this study, most CHM users were adult females, which is compatible with previous reports in which the peak age of the occurrence of eczema was reported to be from 18 to 29 years [50, 51]. The TCM doctors commonly prescribed multiple CHMs with an average of 5.8 CHMs per prescription and more than 90% of the prescriptions containing more than 3 CHMs in this study (Figure 1). This result is similar to our previous work on urticaria (an average of 5.46 CHMs per prescription), and this may be related to the fact that both urticaria and eczema are diseases with a complicated pathogenesis [15].

We propose a CHM network to treat eczema including the commonly used CHMs and their relationships in this study, and this network may be beneficial to understand the complicated CHM treatment model, “TCM pattern identification and treatment,” or “bian-zheng-lun-zhi” in Chinese [14, 19]. “TCM pattern,” or zheng, is a concise summary of the patient's symptoms according to TCM theory, and CHMs are prescribed for the TCM pattern. Due to variety in the patient's manifestations, several TCM patterns may be identified for a single disease, and it may exist to a different extent in each patient. To cope with these combined TCM patterns, TCM doctors usually use several CHMs aimed at certain patterns in one prescription, and thus a CHM pharmacology network is used with the concept of “multiple target, complex diseases” [14, 19]. Therefore, investigating CHM combinations and the construction of a CHM network is more important than studying only one individual CHM. To achieve this goal, SNA was used to graphically demonstrate the CHM network in this study. Modularity, the decisive parameter used to explore clusters, resulted in each cluster of CHMs having strong within-cluster connections and weak between-cluster connections, and this process is similar when prescribing CHMs. TCM doctors usually choose a group of CHMs for the main TCM pattern and then add other CHMs for minor TCM patterns or symptoms. Because of this, SNA has been used to analyze CHM networks for many diseases [15, 16, 28].

Our proposed CHM network for eczema is graphically demonstrated in Figure 2 and may be a valuable reference for TCM doctors when choosing CHMs for certain TCM patterns among eczema patients from the enormous number of available CHMs. When applying the characteristics of the CHM into the CHM network, the TCM pattern-CHM relationships could be seen, even though the information on the TCM pattern of each patient was not provided in the original dataset. The CHM for the wind-dampness-heat pattern constituted the largest cluster, while the heat pattern existed in nearly all of the clusters, which is similar to a previous investigation [52]. The dampness-heat pattern is characterized by skin itching, erythema, local heat, discharge, and swelling in relapse and is usually caused by chronic inflammatory dermatological processes [53, 54]. These manifestations are diagnostic features of eczema, and the high prevalence of this pattern may be related to the wet climate in Taiwan [55]. Moreover, the wind pattern is characterized by rapid movements and swift changes in dermatological symptoms and also presents as the migratory skin lesions around the whole body commonly found among eczema patients. Consequently, combinations of wind, dampness, and heat comprise the majority of TCM patterns among eczema patients and therefore specific CHMs are used for the various combinations of these patterns.

XFS was found to be the most important CHM to treat eczema of all the CHMs, since it was the core of cluster 1 (wind-dampness-heat pattern) and many other CHMs were combined with XFS to effectively treat eczema (Table 4). XFS has been used as a CHM for hundreds of years to expel wind dampness and heat for itching, erythema, and swelling skin lesions with discharge, and its efficacy in treating atopic dermatitis has been proven [56]. In addition, XFS has been reported to have more extensive anti-inflammatory, antiallergic, and antioxidative effects in treating eczema compared with other CHMs used for eczema [45–48]. By modulating the imbalance of Th1 and Th2 cells, XFS can decrease levels of serum IL-4 and interferon gamma which may then correct the constitution of the patient and relieve symptoms [48]. This wide range of action on both pharmacological mechanism and TCM pattern may be the reason why XFS is the most important CHM in the treatment of eczema.

The additive effects of other CHMs combined with XFS are also noteworthy. TCM doctors usually use other CHMs with XFS either to enhance the effectiveness on major pattern or to cover other minor TCM patterns, and these combinations of multiple CHMs can be easily seen in the CHM network diagram. The combination of XFS,* Kochia scoparia *(Di Fu Zi), and* Dictamnus dasycarpus* (Bai Xian Pi) is the major component of cluster 1, and the addition of* Kochia scoparia *(Di Fu Zi) and* Dictamnus dasycarpus* (Bai Xian Pi) may enhance the efficacy of XFS on expelling dampness and heat via enhancing the anti-inflammation and antiallergic effects [39, 44, 57]. In addition,* Lonicera japonica *(Jin Yin Hua) is usually combined with* Forsythia suspensa *(Lian Qiao) and forms the center of cluster 2 to treat the heat pattern of eczema. This combination is also commonly used with cluster 6,* Schizonepeta tenuifolia *(Jing Jie) and* Saposhnikovia divaricata *(Fang Feng), which are well known CHM pair used to expel pathogenic wind. This combination may have combined effects on treating patients with the major heat pattern and minor wind pattern. The unclear pharmacological mechanisms of* Schizonepeta tenuifolia *(Jing Jie) and* Saposhnikovia divaricata *(Fang Feng), especially when used in combination, warrant future studies since* Lonicera japonica *(Jin Yin Hua) and* Forsythia suspensa *(Lian Qiao) already cover nearly all of the pathogenesis of eczema [33–35, 58, 59].

The antimicrobial effect is another interesting finding in the CHM network, in which nearly all clusters had a CHM with antimicrobial effects. Many CHMs with the ability to treat the heat pattern have potent antimicrobial effects, such as Huang-Lian-Jie-Du-Tang,* Forsythia suspensa *(Lian Qiao),* Lonicera japonica *(Jin Yin Hua),* Coix lacryma-jobi* (Yi Yi Ren), and* Dictamnus dasycarpus *(Bai Xian Pi) [35, 60–62]. This finding implies the crucial role of microbial infections in relapsing eczema, which has only been proposed in early reports; however, such infections are not routinely treated due to concerns of drug resistance [63, 64]. Nonetheless, the use of CHM may enhance innate immunity and provide an alternative method to control microbial infections as well as to directly eradicate microbes. For example, Huang-Liang-Jie-Du-Tang has been reported to increase the phagocytic ability of macrophages, and* Coix lacryma-jobi *(Yi Yi Ren) has been reported to increase the number of peripheral cytotoxic T cells and NK cells [60, 62]. Consequently, combining these antimicrobial CHMs with conventional therapy may be a potential therapy to control refractory eczema.

## 5. Conclusion

This study is the first pharmacoepidemiological study to propose a CHM network for the treatment of eczema. Through visualization of the network, core CHMs and CHM combinations for the corresponding TCM patterns and the rationale behind the CHM formulations can clearly be seen. These results of this study can be regarded as a consensus among TCM doctors in Taiwan, and they may therefore provide a valuable reference for both TCM doctors and researchers to select suitable candidates for clinical practice or future studies.

## Figures and Tables

**Table 1:** Age and gender distribution of Chinese herbal medicine (CHM) users (*N* = 132,971).

	CHM users
	*n* (%)
Gender	
Female	83241 (62.6)
Male	49730 (37.4)
Age (years)	
0–10	7092 (5.3)
11–20	27128 (20.4)
21–30	32981 (24.8)
31–40	25905 (19.5)
41–50	18741 (14.1)
51–60	11991 (9.0)
61–70	5153 (3.9)
>70	3980 (3.0)

**Table 2:** The top 5 most commonly prescribed herbal formulas for eczema during 2011 (total prescriptions = 381,282).

Herbal formulas	Ingredients (English name)	Dosage (gm/day)	TCM indications	Number of prescriptions (%)
Xiao-Feng-San (XFS)	*Saposhnikovia divaricata* (Fang Feng), *Atractylodes lancea* (Bai Zhu), *Schizonepeta tenuifolia* (Jing Jie), *Arctium lappa* (Niu Bang Zi), *Glycyrrhiza uralensis* (Gan Cao), *Rehmannia glutinosa* (Sheng Di Huang), *Gypsum Fibrosum* (Shi Gao), *Caulis clematidis armandii*, *Anemarrhena asphodeloides* (Mu Tong), *Angelica sinensis* (Dang Gui), *Cryptotympana pustulata* (Chan Tui), *Sesamum indicum* (Hei Zhi Ma), *Sophora flavescens* (Ku Shen).	4.77	Wind and dampness heat	92075 (24.1)

Qing-Shang-Fang-Feng-Tang	*Saposhnikovia divaricata* (Fang Feng), *Coptis chinensis* (Huang Lian), *Ligusticum chuanxiong* (Chuan Xiong), *Platycodon grandiflorum* (Jie Geng), *Forsythia suspensa* (Lian Qiao), *Scutellaria baicalensis* (Huang Qin), *Mentha haplocalyx* (Bo He), *Angelica dahurica* (Bai Zhi), *Schizonepeta tenuifolia* (Jing Jie), *Glycyrrhiza uralensis* (Gan Cao), *Citrus aurantium* (Zhi Qiao).	4.67	Wind heat and toxin	56903 (14.9)

Xian-Fang-Huo-Ming-Yin	*Lonicera japonica* (Jin Yin Hua), *Saposhnikovia divaricata* (Fang Feng), *Angelica dahurica* (Bai Zhi), *Angelica sinensis* (Dang Gui), *Paeonia lactiflora* (Shao Yao), *Commiphora myrrha* (Mo Yao), *Fritillaria thunbergii* (Zhe Bai Mu), *Trichosanthes kirilowii* (Tian Hua Fen), *Gleditsia sinensis* (Zao Jiao), *Citrus reticulate* (Chen Pi), *Glycyrrhiza uralensis* (Gan Cao).	4.21	Heat toxin	47521 (12.5)

Huang-Lian-Jie-Du-Tang	*Gardenia jasminoides* (Zhi Zi), *Scutellaria baicalensis* (Huang Qin), *Coptis chinensis* (Huang Lian), *Phellodendron chinense* (Huang Bai)	3.91	Heat toxin	41451 (10.9)

Jia-Wei-Xiao-Yao-San	*Paeonia lactiflora* (Shao Yao), *Bupleurum chinense* (Chai Hu), *Atractylodes macrocephala* (Bai Zhu), *Poria cocos* (Fu Ling), *Angelica sinensis* (Dang Gui), *Mentha haplocalyx* (Bo He), *Glycyrrhiza uralensis* (Gan Cao), *Zingiber officinale* (Gan Jiang), *Paeonia suffruticosa* (Mu Dan Pi), *Gardenia jasminoides* (Zhi Zi).	4.13	Liver qi stagnation with heat, spleen qi deficiency	36423 (9.6)

**Table 3:** The top 10 most commonly used single herbs for eczema during 2011 (total prescriptions = 381,282).

Single herb Latin name	English name	Dosage (gm/day)	TCM indications	Number of prescriptions (%)
*Forsythia suspensa *	Lian Qiao	1.20	Heat toxin	64868 (17.0)
*Coix lacryma-jobi *	Yi Yi Ren	1.34	Dampness, spleen qi deficiency, and heat	48581 (12.7)
*Taraxacum mongolicum *	Pu Gong Ying	1.30	Heat	16101 (12.1)
*Dictamnus dasycarpus *	Bai Xian Pi	1.26	Dampness and heat	41897 (11.0)
*Lonicera japonica *	Jin Yin Hua	1.21	Heat toxin	41895 (11.0)
*Glycyrrhiza uralensis *	Gan Cao	0.87	Spleen qi deficiency, moderation of properties of other drugs	39814 (10.4)
*Rheum palmatum *	Da Huang	1.34	Heat toxin and blood heat	38194 (10.0)
*Paeonia suffruticosa *	Mu Dan Pi	1.38	Blood heat, stasis	33929 (8.9)
*Kochia scoparia *	Di Fu Zi	1.56	Heat	33417 (8.8)
*Smilax glabra *	Tu Fu Ling	1.41	Dampness and heat	32565 (8.5)

**Table 4:** Top 10 pairs of Chinese herbal medicines (CHMs) used in combination for eczema.

CHM A (English name)		CHM B (English name)	Instances	Prevalence (%)
*Lonicera japonica *(Jin Yin Hua)	With	*Forsythia suspensa *(Lian Qiao)	18873	5.0
Xiao-Feng-San	With	*Dictamnus dasycarpus *(Bai Xian Pi)	18293	4.8
*Taraxacum mongolicum *(Pu Gong Ying)	With	*Forsythia suspensa *(Lian Qiao)	14816	3.9
*Dictamnus dasycarpus *(Bai Xian Pi)	With	*Kochia scoparia* (Di Fu Zi)	14001	3.7
Xiao-Feng-San	With	*Kochia scoparia* (Di Fu Zi)	13993	3.7
*Coix lacryma-jobi* (Yi Yi Ren)	With	*Kochia scoparia* (Di Fu Zi)	13390	3.5
Qing-Shang-Fang-Feng-Tang	With	*Taraxacum mongolicum* (Pu Gong Ying)	12776	3.4
Xiao-Feng-San	With	*Forsythia suspensa *(Lian Qiao)	11980	3.1
Xiao-Feng-San	With	*Coix lacryma-jobi* (Yi Yi Ren)	11900	3.1
Xian-Fang-Huo-Ming-Yin	With	*Forsythia suspensa *(Lian Qiao)	11065	2.9

**Table 5:** Possible pharmacological mechanisms of Chinese herbal medicines (CHMs) used for eczema.

CHM	Possible mechanisms
**Single herb (SH)**	
*Forsythia suspensa *(Lian Qiao)	Antioxidation [32] Anti-inflammation [33] Antiallergy effect [34] Antibacterial effect [35]
*Coix lacryma-jobi* (Yi Yi Ren)	Anti-inflammation/antiallergic effect [36]
*Taraxacum mongolicum *(Pu Gong Ying)	Antioxidation [37]
*Dictamnus dasycarpus *(Bai Xian Pi)	Antiallergic effect [38] Anti-inflammation [39]
*Lonicera japonica *(Jin Yin Hua)	Anti-inflammation [40] Antiallergic effect [41]
*Glycyrrhiza uralensis *(Gan Cao)	Anti-inflammation/antioxidation [42]
*Rheum palmatum *(Da Huang)	Antiallergic effect [43]
*Paeonia suffruticosa *(Mu Dan Pi)	Antiallergic effect [41]
*Kochia scoparia *(Di Fu Zi)	Antiallergic effect [44]

**Herbal formula (HF)**	
Xiao-Feng-San	Antiallergic effect [45, 46] Antioxidation/anti-inflammation [47] Immunomodulation of Th1/Th2 balance [48]
Huang-Lian-Jie-Du-Tang	Anti-inflammation, decreasing cellular adhesion molecule expression [49] Immunomodulation of Th1/Th2 balance [48]
